# Reliability and Concurrent Validity of the Likert‐Type Stuttering Severity Scale and the Visual Analog Scale in Turkish‐Speaking Adults Who Stutter

**DOI:** 10.1111/1460-6984.70325

**Published:** 2026-08-31

**Authors:** Ayşe İlayda Mutlu, İlkem Uçal

**Affiliations:** ^1^ Speech and Language Therapy Department Faculty of Health Sciences Başkent University Ankara Turkiye; ^2^ Speech and Language Therapy Department, Faculty of Health Sciences Ankara Yıldırım Beyazıt University Ankara Turkiye

**Keywords:** 11‐point Severity Scale, reliability, stuttering severity, validity, Visual Analog Scale

## Abstract

**Purpose:**

This study examined the test–retest and inter‐rater reliability of non‐clinician severity judgments using two brief rating tools—the 11‐point Severity Scale (SEV) and the Visual Analog Scale (VAS)—and evaluated their concurrent validity with clinician‐rated Stuttering Severity Instrument‐4‐Turkish version (SSI‐4‐TR) scores.

**Method:**

This observational cross‐sectional study used speech samples from 15 adults who stutter as stimulus materials. Recordings were evaluated by two speech‐language therapists (SLTs) and two independent groups of non‐clinician raters (*n* = 26 per group). SLTs assessed stuttering severity using the SSI‐4‐TR. One non‐clinician group rated severity using a VAS, whereas the other used SEV. All ratings were completed online across three sessions. To examine temporal stability, 30% of the samples were re‐rated after a one‐week interval.

**Results:**

Both tools demonstrated excellent test–retest reliability, with intraclass correlation coefficients of 0.997 for the VAS and 0.987 for the SEV. Inter‐rater reliability was moderate, with ICC values of 0.656 for the VAS and 0.573 for the SEV. Both scales showed strong positive correlations with SSI‐4‐TR scores, with correlation coefficients of 0.835 for the VAS and .814 for the SEV (*p* < 0.001).

**Conclusions:**

The findings indicate that the VAS and SEV demonstrated high test–retest reliability and moderate inter‐rater reliability and showed strong associations with SSI‐4‐TR scores, supporting the use of brief perceptual rating scales as practical screening instruments in large‐scale and time‐limited clinical and research settings where rapid and resource‐efficient assessment is required.

**WHAT THIS PAPER ADDS:**

*What is already known on this subject*

- Perceptual severity ratings are widely used to estimate stuttering severity in both clinical and research contexts. Likert‐type scales have demonstrated acceptable reliability when applied by trained listeners, whereas the Visual Analog Scale (VAS) has shown strong psychometric performance in several perceptual domains such as voice and resonance assessment. However, empirical evidence regarding the reliability and validity of a single‐item VAS for evaluating stuttering severity remains limited. In addition, it is not yet clear whether trained non‐clinician raters can apply brief perceptual severity scales consistently, particularly when ratings are compared with standardized clinical measures such as the SSI‐4.

*What this study adds to the existing knowledge*

- This study provides novel psychometric evidence comparing an 11‐point Likert‐type severity scale (SEV) and a Visual Analog Scale (VAS) for rating stuttering severity using trained non‐clinician raters. Both tools demonstrated excellent test–retest reliability, moderate inter‐rater reliability, and strong concurrent validity with clinician‐rated SSI‐4‐TR scores. The findings extend previous research by demonstrating that brief perceptual severity ratings can produce stable and clinically meaningful estimates when administered by trained but non‐expert listeners. The study also contributes to the literature by directly comparing continuous and categorical response formats within the same experimental framework.

*What are the clinical implications of this study?*

- The findings suggest that brief perceptual severity scales such as VAS and SEV may serve as practical adjunct tools for screening, monitoring change, and large‐scale data collection in clinical and research settings where time and resources are limited. Trained non‐clinician raters may provide consistent evaluations under controlled conditions, supporting their potential role in structured assessment contexts. However, perceptual ratings should not replace comprehensive clinical evaluation and may be most informative when used alongside standardized measures such as the SSI‐4 or frequency‐based indices to obtain a more complete representation of stuttering severity.

## 1 Introduction

Stuttering is a neurodevelopmental speech disorder characterized by disruptions in the fluency and rhythm of speech. Beyond overt speech disfluencies, stuttering is a condition shaped by individual, environmental, and social factors, and its severity varies substantially across individuals and communicative contexts (Ambrose et al. 2015; Ortiz‐Alvarez and Arenas 2025; Tichenor and Yaruss 2019). Although stuttering is widely recognized as a multifactorial condition, clinical and research contexts frequently require the estimation of severity as perceived from speech samples, as this information contributes to clinical decision‐making, treatment planning, and monitoring of change over time (De Sonneville‐Koedoot et al. 2015; Chow et al. 2023). The assessment of stuttering severity plays a central role in clinical decision‐making, treatment planning, and outcome monitoring (Venkatesan 2025). Importantly, severity is not defined solely by the frequency of stuttering‐like disfluencies but also by temporal features, observable physical concomitants, and listeners’ global impressions of speech effort, struggle, and impact (Brundage et al. 2021). As such, stuttering severity inherently involves perceptual judgment.

The Stuttering Severity Instrument (SSI) is a widely used clinical measure for assessing the severity of stuttering, developed over 37 years and currently in its fourth version (SSI‐4) (Riley 2009). The scale assesses the severity of stuttering across three main parameters: frequency of stuttering, duration of stuttering, and physical concomitant behaviours. The scores obtained from these subtests are combined to calculate a total severity score, and individuals are categorized between “very mild” and “very severe.” The SSI‐4 is frequently used as a reference measure in both clinical practice and research (Riley 2009). Despite its widespread use, the conceptualization and measurement of stuttering severity remain debated, and concerns have been raised regarding the reliability and interpretability of certain SSI components, particularly when fine‐grained changes in behaviour are of interest (Davidow 2021; Davidow and Scott 2017).

Nevertheless, the SSI‐4 continues to serve as a commonly used clinical reference point for quantifying stuttering severity in both research and applied settings. Nonetheless, the conceptualization and quantification of stuttering severity continue to be a contentious issue (Davidow and Scott 2017; Hall et al. 1987; Lewis 1995). Various limitations have been reported, including multi‐component scoring procedures, subjective judgments in assessing physical accompanying symptoms, and the inability of composite scores to adequately reflect the experiential and perceptual dimensions of severity (Davidow 2021; Onslow et al. 1992; Tichenor and Yaruss 2019). Moreover, the intensity of stuttering is progressively regarded as a dynamic phenomenon that fluctuates based on the listener and context, rather than a static and speech‐specific trait (Ortiz‐Alvarez and Arenas 2025). This situation illustrates the importance of complementary assessment approaches that more directly reflect the severity and are applicable for repeated and ecologically valid measurements. Perceptual severity rating scales are commonly employed in clinical and research settings to assess overall perceptions of stuttering severity.

### 1.1 Severity Scales

The Likert‐type stuttering severity rating scale is a prominent tool for the subjective assessment of stuttering severity, supported by studies demonstrating its reliability in listener ratings after minimal training, and commonly used in both clinical practice and research settings (Eve et al. 1995; O'Brian et al. 2004). These scales are also referred to as equal‐appearing interval (EAI) scales, in which listeners assign numerical values anchored by fixed endpoints representing the least and most severe stuttering. This method involves numerically ordered response categories that are often treated as ordinal in measurement terms, on which listeners assign a numerical value to a given stimulus. These scales use fixed endpoints and whole numbers. In clinical and research settings, Likert‐type scales with varying numbers of fixed response categories are commonly used for evaluating stuttering severity. The most severe stuttering is at one end of the scale, and the least severe stuttering is at the other end. Listeners are expected to assign a numerical value that reflects their overall perception of the stuttering severity (O'Brian et al. 2004). When using perceptual stuttering severity rating scales, it is assumed that listeners consider various aspects, such as the rate of stuttering, its frequency, and the intensity of individual stuttering moments (O'Brian et al. 2004). These scales can be used in conjunction with other stuttering severity assessment tools and serve as self‐assessment instruments for individuals rating their speech in daily life contexts. Parents can also use them to rate their children's speech in real‐life situations (Carey et al. 2014; Johnson et al. 2025). Despite their utility, Likert‐type scales have some inherent psychometric constraints. Raters may not perceive the intervals between response options as equal, as they produce ordinal data (Emerson 2017). This characteristic may reduce measurement sensitivity, particularly when evaluating continuous phenomena such as stuttering severity, which can vary along a gradient rather than in discrete categories (O'Brian et al. 2004).

Additionally, the restricted number of fixed points may result in ceiling or floor effects, particularly in repeated‐measure designs or when identifying subtle therapeutic changes (Lee and Paek 2014). As a result, researchers in related fields have looked into more continuous and detailed perceptual methods (Grant et al. 1999). One such approach is the VAS, which provides a linear continuum to capture subtle variations in perceived severity and potentially improve measurement precision.

### 1.2 Visual Analog Scale

The Visual Analog Scale (VAS) consists of a continuous 100 mm line on which participants mark the perceived intensity of a condition. It is widely used in clinical and perceptual assessments and also considered valuable because it allows for the fine‐grained capture of perceptual differences (Hasson and Arnetz 2005). In speech and language research, the VAS has also been successfully applied in the assessment of hypernasality, audible nasal airflow, speech intelligibility, and voice disorders and has been reported to show strong reliability and validity in these perceptual domains (Bettens et al. 2018; Castick et al. 2017; Lee et al. 2018; Yu et al. 2002). Despite its theoretical advantages and widespread use in other perceptual domains, empirical evidence shows that the psychometric performance of a single‐item VAS for rating stuttering severity is limited. In particular, evidence regarding its test–retest reliability, inter‐rater reliability, and concurrent validity relative to commonly used clinical severity indicators is scarce. Moreover, it remains unclear whether individuals without extensive clinical experience can apply the VAS consistently when evaluating stuttering severity.

### 1.3 The Present Study

A review of the literature indicates that findings comparing VAS and Likert‐type scales are inconsistent. Although VAS may demonstrate greater sensitivity in some contexts, it does not consistently provide superior reliability or measurement precision compared to well‐structured Likert scales. Psychometric and simulation studies indicate that measurement improvement levels off after about six to seven response categories, and enhancing scale resolution may diminish reliability by adding measurement error (Simms et al. 2019). Similarly, studies comparing continuous and categorical response formats across psychological and health domains have reported comparable reliability and validity under certain conditions, indicating that scale performance may depend on contextual and methodological factors (Kuhlmann et al. 2017). Evidence regarding sensitivity also varies across domains. For example, VAS and Likert‐type scales show similar responsiveness in some clinical outcomes such as lung disease and osteoarthritis (Bolognese et al. 2003; Guyatt et al. 1987), whereas VAS may demonstrate lower ceiling effects or improved sensitivity near extreme values in patient satisfaction and mood assessment contexts (Haslbeck et al. 2025; Voutilainen et al. 2016). Importantly, the perceptual nature of stuttering severity has been discussed within psychophysical frameworks that distinguish between prothetic (graded) and metathetic (qualitative) continua (Stevens 2017). Early work suggested that perceived stuttering severity may function as a graded continuum, raising questions about whether discrete category‐based formats fully capture perceptual differences (Schiavetti et al. 1983). However, the present study does not aim to determine the psychophysical structure of stuttering severity itself. Rather, this theoretical perspective provides a conceptual framework for examining whether different response formats—such as a continuous VAS and a discrete numerical scale—demonstrate comparable psychometric performance in practical rating contexts. Recent findings further suggest that equal‐appearing interval approaches may yield meaningful severity judgments under specific methodological conditions, highlighting the importance of stimulus characteristics and rater familiarization procedures (Johnson et al. 2026). Despite its theoretical suitability and demonstrated psychometric utility in related perceptual domains, the reliability and validity of a single‐item VAS for rating stuttering severity have not been systematically examined. Moreover, there is a practical need to determine whether trained but non‐clinician individuals (e.g., senior students, clinical assistants, or research personnel) can provide consistent perceptual severity ratings, as such groups may contribute to large‐scale or repeated assessments in both research and applied contexts. Accordingly, this study aimed to evaluate the psychometric properties of two brief perceptual rating tools (SEV and VAS) by examining (a) test–retest reliability, (b) inter‐rater reliability, and (c) concurrent validity with clinician‐rated SSI‐4‐TR scores in non‐clinician raters.

## 2 Method

### 2.1 Research Design

This observational, cross‐sectional psychometric study evaluated the reliability and concurrent validity of two perceptual stuttering severity rating tools, VAS and SEV, administered by non‐clinician raters. A video‐based procedure standardized speech sample presentation and allowed repeated viewing for reliability analyses. The design included test–retest reliability, inter‐rater reliability, and correlational analyses with clinician‐derived SSI‐4‐TR scores. Ethical approval was obtained from the Lokman Hekim University Scientific Research Ethics Committee (Decision date: 28 February 2025; Decision No: 2025/026; Study Code No: 2025011). Digital informed consent was obtained from all participants before participation.

### 2.2 Participants

#### 2.2.1 Raters

A total of 54 raters (30 women, 24 men) were initially recruited to listen to and evaluate speech samples from adults who stutter. All participants completed a brief demographic questionnaire and provided digital informed consent before participation. The raters were final‐year undergraduates enrolled in speech and language therapy programs at universities in Ankara. All had completed the courses Assessment and Therapy in Fluency Disorders, which included supervised clinical experience in stuttering assessment and intervention. Recruitment was conducted through an online volunteer announcement. Participant data from individuals reporting a history of speech, language, hearing, neurological, or developmental disorders were not included in the analyses. Therefore, one non‐native Turkish speaker and one participant reporting a speech/hearing impairment were not included in the analyses, resulting in a final sample of 52 raters. These raters were selected to represent trained but non‐clinician listeners who possess foundational academic knowledge and supervised clinical exposure to fluency disorders but have not yet begun independent professional practice. The sample size was established in accordance with prior studies on perceptual reliability, which generally encompass around 20–30 raters (e.g., Eve et al. 1995; O'Brian et al. 2004).

#### 2.2.2 Speakers

Fifteen adults who stutter (7 female, 8 male), aged between 18 and 32 years (M = 24.7, SD = 3.8) participated in the study. Participants were recruited through speech‐language therapists working in multiple hospitals and through the Turkish Stuttering Association (Kekemeler Derneği). The diagnosis of stuttering was established by certified speech‐language therapists based on client self‐report, case history information, and direct clinical observation of stuttering behaviours. Inclusion criteria required participants to be native Turkish speakers with bilaterally normal hearing, adequate literacy skills, and no known intellectual, cognitive, or neurological disorders. Participants exhibited a spectrum of stuttering severities to guarantee variability in perceptual evaluations. All participants provided informed consent before participation, and no participants were excluded after recruitment.

The study included 15 speakers to make sure there was a range of stuttering severity levels. In perceptual reliability studies, the precision of reliability estimates is contingent not solely on the quantity of speakers but also on the number of raters (Saito et al. 2006). In this study, the fact that each speech sample was evaluated by a relatively large group of raters increases the reliability of the estimates. Additionally, the heterogeneity of participants’ stuttering severity levels allowed the validity analyses to be examined across a broad severity range.

### 2.3 Procedure

#### 2.3.1 Speech Stimuli

Assessment sessions were conducted individually by the first author with adults who stutter. Each session lasted approximately 20 min and included two speaking tasks: a spontaneous monologue and a standardized reading passage. For each task, segments were selected beginning 30 s after task onset to minimize potential adaptation effects and to capture representative speech performance. The assessments were given online through Zoom, and before the data collection, the participants filled out a demographic questionnaire.

For the monologue task, participants responded to open‐ended prompts to elicit approximately 300–500 syllables of spontaneous speech. They then read the 256‐syllable standardized passage from the SSI‐4‐TR. All sessions were video recorded under standardized acoustic conditions to ensure comparable recording quality. Participants were instructed to complete recordings in a quiet environment using personal computers with built‐in microphones or headsets, maintaining a consistent speaking distance from the microphone. Audio levels were monitored during recording to ensure adequate signal clarity and minimize background noise. For the analysis, 10 min of speech from each participant—consisting of one monologue segment and one reading segment—were chosen as representative samples. Two certified speech‐language therapists with more than five years of clinical experience in the assessment and treatment of fluency disorders independently evaluated all recordings using the SSI‐4‐TR. Before scoring, clinicians received standardized training and completed practice ratings to ensure consistency. These clinician‐derived scores served as the reference measure for subsequent validity analyses. For the perceptual rating phase, the selected samples were randomly ordered and separated by short silent intervals to minimize carry‐over effects. The recordings were compiled into three rating files, each containing five samples, and used as stimulus materials for the SEV and VAS procedures.

#### 2.3.2 SEV/VAS Rating Procedure

Fifty‐two raters were randomly assigned to one of two groups (*n* = 26 each) to evaluate stuttering severity using either the SEV or VAS scale. Randomization was stratified by gender to ensure comparable gender distributions across groups. All ratings were completed individually and independently online using personal computers and headphones following standardized instructions. Before data collection, raters received standardized written instructions describing the task and the perceptual dimensions to be considered (e.g., frequency, duration, and overall speech effort). Instruction clarity was assessed using a five‐point Likert‐type scale. To enhance consistency across raters, each group participated in a brief orientation session that included three anchor recordings representing mild, moderate, and severe stuttering, along with two additional practice samples. These recordings were used solely for calibration and were not included in the analyses. Video stimuli were delivered individually through online experimental platform. Speech samples were presented in mp4 format and viewed sequentially. Each recording played automatically to completion, and backtracking to previously viewed samples was disabled to prevent comparative judgments. The stimulus presentation order was randomized for each participant and counterbalanced across raters to minimize order and carryover effects. For the VAS group, severity was indicated on a 100‐mm horizontal line anchored by “no stuttering” and “very severe stuttering,” and scores were recorded as continuous millimetre values. For the SEV group, raters selected a value on an 11‐point numerical scale ranging from 0 (“no stuttering”) to 10 (“very severe stuttering”). An 11‐point scale (0–10) was selected to allow inclusion of a true zero point representing the absence of stuttering and to maintain conceptual consistency with the continuous VAS format. Because the task required sustained attention, ratings were completed across three sessions with short breaks to minimize fatigue. All three sessions were completed within a single day. To evaluate intra‐rater reliability, five speech samples, constituting approximately one‐third of the total stimulus set, were randomly selected and presented to all raters in the second evaluation session. This repeated evaluation was conducted using a randomly selected subsample of the existing speech samples instead of the entire stimulus set, with the aim of determining the stability of perceptual evaluations over time. This approach is consistent with methodological recommendations that suggest repeated reliability assessments may not require re‐evaluation of the entire sample (Alpar 2011; Anthoine et al. 2014; Mokkink et al. 2022). These samples were re‐presented after a one‐week interval. A one‐week interval was selected to minimize recall bias, consistent with methodological recommendations for test–retest reliability studies (De Vet et al. 2011) and previous methodological and systematic review evidence (Marx et al. 2003; Park et al. 2018). To minimize potential sources of bias, all raters were blinded to clinician‐derived SSI‐4‐TR scores and completed

### 2.4 Data Analysis

All Statistical Analyses Were Conducted Using IBM SPSS Statistics (Version 29). Descriptive Statistics Were Calculated for all Demographic and Rating Variables. Reliability Was Evaluated through Inter‐rater and Test–retest Analyses. Inter‐rater Reliability Was Examined Using Intraclass Correlation Coefficients (ICCs; Two‐way Random Model, Absolute Agreement). Validity Was Assessed through Spearman Correlation Analyses among SEV, VAS, and SSI‐4‐TR Scores. A Significance Level of *p* < 0.01 Was Adopted for all Analyses. No Missing Data Were Observed, and all Raters Completed all Assigned Rating Sessions.

## 3 Results

Twenty‐six raters used the SEV, and twenty‐six used the VAS. Each group consisted of 16 female and 10 male raters. Participants in the SEV group were aged 19–23 years (M = 22.4 years), whereas participants in the VAS group were aged 20–24 years (M = 22.5 years). Comprehensibility of the task instructions was high in both groups. In the SEV group, 96.1% of raters reported fully understanding the instructions (“Agree” = 82.7%; “Somewhat agree” = 13.4%), whereas 3.9% indicated partial understanding (“Somewhat disagree” = 3.9%; “Disagree” = 0%). In the VAS group, 92.3% of raters said they fully understood, with 78.9% saying they “Agree” and 13.4% saying they “Somewhat agree.” A small number of raters (5.1% “Somewhat disagree” and 2.6% “Disagree”) said they only partially understood. These findings demonstrate that the raters clearly understood and consistently applied the rating procedures for both scales. The SSI‐4‐TR results for the speakers included in the analysis are presented in Table 1.

**TABLE 1:** Descriptive results of SSI‐4‐TR for adults who stutter.

Participant (*n*)	Sex	Age (years)	SSI‐4‐TR total score	Severity equivalent	Percentile rank
1	Male	26	33	Severe	89–95
2	Male	33	20	Mild	24–40
3	Female	26	18	Mild	24–40
4	Male	23	15	Mild	12–23
5	Female	28	22	Moderate	41–60
6	Female	25	30	Severe	78–88
7	Male	32	19	Mild	24–40
8	Male	21	14	Mild	12–23
9	Female	24	7	Very mild	1–4
10	Female	21	11	Mild	12–23
11	Male	23	36	Very severe	96–99
12	Female	23	38	Very severe	96–98
13	Male	26	24	Moderate	61–77
14	Male	18	16	Mild	24–40
15	Female	30	17	Mild	24–40

Abbreviations: *n*, participant number; SSI‐4‐TR, Stuttering Severity Instrument–Fourth Edition, Turkish Version.

### 3.1 Reliability

#### 3.1.1 Test–Retest Reliability

The first research question addressed the test–retest reliability of the rater evaluations. As shown in Table 2, both SEV and VAS demonstrated excellent reliability across repeated measurements. The VAS yielded an ICC of .997, and the SEV yielded an ICC of .987.

**TABLE 2:** Test–retest reliability of SEV and VAS ratings.

Scale	ICC	95% CI	*p*
VAS	0.997	0.996–0.998	< 0.001
SEV	0.987	0.982–0.990	< 0.001

Abbreviations: CI = confidence interval; ICC = intraclass correlation coefficient; SEV = 11‐point Severity Scale; VAS = Visual Analog Scale.

#### 3.1.2 Inter‐Rater Reliability

Table 3 shows the intraclass correlation coefficients (ICCs) for the SEV and VAS ratings. Visual inspection of the coefficients indicates moderate inter‐rater reliability for both scales, with the VAS demonstrating slightly higher agreement than the SEV. The ICC for the VAS was .656, whereas the ICC for the SEV was .573.

**TABLE 3:** Inter‐rater reliability of SEV and VAS ratings.

Scale	ICC	95% CI	*p*
VAS	0.656	0.43–0.82	< 0.001
			
SEV	0.573	0.29–0.78	< 0.001
			

Abbreviations: CI = confidence interval; ICC = intraclass correlation coefficient; SEV = 11‐point Severity Scale; VAS = Visual Analog Scale.

### 3.2 Validity

Subsequent analyses examined the validity of SEV and VAS ratings relative to clinician‐based SSI‐4‐TR scores. As presented in Table 4, both SEV and VAS demonstrated strong and statistically significant positive correlations with SSI‐4‐TR scores, indicating that perceptual ratings provided by non‐clinician raters closely aligned with clinician‐based assessments.

**TABLE 4:** Correlations between SEV/VAS and SSI‐4‐TR scores.

Scale—SSI‐4‐TR	Spearman's ρ	95% CI	*p*
VAS—SSI‐4‐TR	0.835	0.802–0.864	< 0.001
SEV—SSI‐4‐TR	0.814	0.777–0.846	< 0.001

Abbreviation: CI, confidence interval.

## 4 Discussion

In this study, the reliability and concurrent validity of SEV and VAS in evaluating stuttering severity by non‐clinician raters were examined. The findings indicated that both instruments demonstrated high test–retest reliability, moderate inter‐rater reliability, and strong concurrent validity.

The first finding concerns the test–retest reliability of the two scales. The VAS and SEV both showed great consistency, with ICC values of .997 and .987, respectively. According to Koo and Li (2016), ICC values above 0.90 indicate excellent reliability. 90 indicates excellent reliability, confirming that both tools produced highly stable results within raters across sessions (Koo and Li 2016). Consistent with these results, previous studies have shown that non‐clinician raters can achieve substantial reliability in perceptual stuttering assessments when provided with clear rating instructions and minimal training (Eve et al. 1995; Knight and Keith 2005). Together, these findings suggest that trained non‐clinician raters may provide consistent perceptual evaluations under controlled conditions and may be useful in research and structured assessment contexts.

ICC values indicated moderate inter‐rater reliability for the VAS, whereas the SEV yielded slightly lower agreement. These results indicate that both scales capture similar aspects of stuttering severity. The continuous format of the VAS may allow finer gradation of perceptual judgments; however, reliability levels were broadly comparable across the two instruments. Previous perceptual reliability studies have reported similar findings. Martin and Haroldson (1992) discovered that non‐clinician raters exhibited restricted yet quantifiable intra‐ and inter‐rater reliability while employing a 9‐point severity scale (Martin and Haroldson 1992). Onslow et al. (1992) further emphasized the importance of structured training to enhance reliability in perceptual judgments (Onslow et al. 1992). In the voice‐disorders literature, comparable variability among raters has been observed (Englert et al. 2021). Collectively, these results confirm the present study's findings, bolstering the idea that continuous scales and guided familiarization enhance perceptual rating reliability (Englert et al. 2021).

The strong correlations observed between SSI‐4‐TR and both VAS (*r* = .835) and SEV (*r* = .814) indicate a substantial degree of association while also suggesting that these measures may reflect related, though not identical, aspects of stuttering severity. Given that the SSI‐4 (Riley 2009) is one of the most widely used norm‐referenced instruments for evaluating stuttering severity, the observed associations provide additional evidence supporting the concurrent validity of brief perceptual severity ratings. Furthermore, the Turkish adaptation has demonstrated strong psychometric properties (Mutlu et al. 2025), strengthening the interpretability of the present findings. Previous research comparing brief Likert‐type severity ratings with standardized assessments has yielded mixed results. For example, Davidow (2021) said that the SSI‐4 was more reliable than a 5‐point Global Severity Rating Scale. This means that simplified global ratings may not be good enough to replace more detailed measures. In contrast, O'Brian et al. (2004) found strong agreement between a 9‐point severity scale and percentage of stuttered syllables (%SS), indicating that perceptual ratings can provide reliable and valid estimates of stuttering severity. Consistent with this latter line of evidence, the present findings support the use of brief ratings such as VAS and SEV as efficient yet psychometrically sound alternatives to more time‐intensive standardized assessments, particularly in screening or large‐scale clinical and research settings. Nevertheless, combining perceptual severity ratings with frequency‐based measures may offer the most comprehensive representation of stuttering severity, especially in speech samples characterized by disproportionate frequency–severity patterns.

Prior research has debated whether Likert‐type scales are psychometrically appropriate for capturing perceived stuttering severity, with early work suggesting a prothetic continuum and a curvilinear relationship between interval scaling and direct magnitude estimation (DME) (Schiavetti et al. 1983). More recent evidence, however, indicates that EAI scales may still provide valid severity judgments under certain methodological conditions, highlighting that scale performance may depend on stimulus selection and rater instructions/training (Johnson et al. 2026). Extending this literature, the present study evaluated two clinically feasible single‐item formats (a 100‐mm VAS and an 11‐point numerical SEV scale) completed by trained non‐clinician raters and demonstrated excellent test–retest reliability, moderate inter‐rater reliability, and strong concurrent validity with clinician‐rated SSI‐4‐TR scores.

### 4.1 Limitations

This study has several limitations. First, the non‐clinician raters were exclusively undergraduate health sciences students specializing in speech and language therapy; consequently, the findings may not apply to individuals with diverse educational backgrounds or varying levels of clinical experience. Because these raters had prior academic exposure to fluency disorders, their performance may not fully represent judgments made by completely untrained listeners. Second, the speech samples included only adults who stutter, excluding preschool and school‐aged populations. Subsequent research ought to incorporate developmental samples to ascertain the generalizability of the identified psychometric patterns across various age cohorts. Third, all evaluations were conducted online via participants’ personal computers and headphones, which may have introduced variability in audio quality and listening conditions. Finally, perceptual severity ratings are inherently subjective and may be influenced by individual differences in listeners’ internal criteria or response tendencies, which could contribute to variability in inter‐rater reliability.

### 4.2 Conclusions

The present study demonstrated that both the VAS and the SEV provide high test–retest reliability and moderate inter‐rater reliability and strong concurrent validity for estimating stuttering severity when administered by trained non‐clinician raters. These findings also suggest that such tools may be meaningfully applied by trained but non‐expert individuals, including clinical assistants, students, or research personnel, particularly in controlled research settings. Both measures showed high test–retest reliability and moderate inter‐rater reliability and exhibited excellent correlations with clinician‐administered SSI‐4‐TR scores, supporting their concurrent validity. These findings indicate that brief severity ratings closely reflect standardized clinical assessments even when completed by individuals without extensive clinical experience. Importantly, to the authors’ knowledge, empirical psychometric evidence regarding the use of a single‐item VAS for rating stuttering severity has been limited. Therefore, the present findings provide novel evidence supporting the reliability and validity of the VAS as a practical and time‐efficient assessment tool. Because they are easy to use, practical, and perform well, VAS and SEV can be helpful additional tools alongside more detailed standardized tests, especially in situations like screenings, large assessments, and research or community settings where time and resources are tight. Nonetheless, the integration of perceptual severity ratings with frequency‐based measures may yield the most thorough depiction of stuttering severity, particularly in speech samples exhibiting disproportionate frequency–severity patterns. Overall, the results suggest that using short severity rating tools in both clinical settings and research can help make stuttering assessments quicker, more reliable, and more relevant to real‐life situations.

### 4.3 Future Directions

Future research should build on these findings in several ways. First, direct comparisons between clinician and non‐clinician raters on SEV and VAS scores would clarify the extent to which professional expertise influences perceptual judgments of stuttering severity. Second, experimental designs could evaluate the effect of structured rater training by comparing trained and untrained groups, thereby identifying how familiarity with rating procedures impacts reliability and validity.

## Funding

This research did not receive support from any funding agency or industry.

## Conflicts of Interest

The authors declare no conflicts of interest.

## Data Availability

The data that support the findings of this study are available from the corresponding author upon reasonable request.
